# Inhibitory synapse dysfunction and epileptic susceptibility associated with KIF2A deletion in cortical interneurons

**DOI:** 10.3389/fnmol.2022.1110986

**Published:** 2023-01-17

**Authors:** Nuria Ruiz-Reig, Dario García-Sánchez, Olivier Schakman, Philippe Gailly, Fadel Tissir

**Affiliations:** ^1^Institute of Neuroscience, Université catholique de Louvain, Brussels, Belgium; ^2^College of Health and Life Sciences, Hamad Bin Khalifa University, Doha, Qatar

**Keywords:** epilepsy, interneuron, tangential migration, microtubules, inhibitory synapses

## Abstract

Malformation of cortical development (MCD) is a family of neurodevelopmental disorders, which usually manifest with intellectual disability and early-life epileptic seizures. Mutations in genes encoding microtubules (MT) and MT-associated proteins are one of the most frequent causes of MCD in humans. KIF2A is an atypical kinesin that depolymerizes MT in ATP-dependent manner and regulates MT dynamics. In humans, single *de novo* mutations in KIF2A are associated with MCD with epileptic seizures, posterior pachygyria, microcephaly, and partial agenesis of corpus callosum. In this study, we conditionally ablated KIF2A in forebrain inhibitory neurons and assessed its role in development and function of inhibitory cortical circuits. We report that adult mice with specific deletion of KIF2A in GABAergic interneurons display abnormal behavior and increased susceptibility to epilepsy. KIF2A is essential for tangential migration of cortical interneurons, their positioning in the cerebral cortex, and for formation of inhibitory synapses *in vivo*. Our results shed light on how KIF2A deregulation triggers functional alterations in neuronal circuitries and contributes to epilepsy.

## 1. Introduction

Malformations of cortical development (MCD) are characterized by the interruption of one or more developmental processes in the cerebral cortex, such as proliferation, migration and/or connectivity of neural cells (Parrini et al., 2016; Hakanen et al., 2019). In humans, MCD commonly cause developmental delay, intellectual disability, and epilepsy. Most MCD patients develop epileptic seizures in the first year of life, and 40% of childhood epilepsies are intractable or drug-resistance (Barkovich et al., 2012). Mutations in genes implicated in radial migration of glutamatergic neurons in the cortex can cause lissencephaly or “smooth brain,” a neuronal migration disorder that affects the organization and lamination of the cortex. Lissencephaly has different degrees of severity, including agyria (absence of gyri in the brain surface) and pachygyria (decreased number and broader gyri). On the other hand, defects in the differentiation, tangential migration, positioning or connectivity of cortical interneurons underlie interneuronopathies, a group of disorders associated with childhood epilepsy and neurodevelopmental disorders such as autism spectrum and schizophrenia (Lodato et al., 2011; Katsarou et al., 2017; Laclef and Metin, 2018). Many cytoskeletal proteins are implicated in both types of migration, radial and tangential migration, and hence, mutations in their genes could result in lissencephaly and interneuronopathies (Stouffer et al., 2016). Therefore, when MCD is due to mutation in cytoskeleton-related genes, it is not clear whether epilepsy arises from defects in cortical lamination, aberrant migration of GABAergic interneurons, or both.

KIF2A is an atypical kinesin widely expressed in the nervous system. It is believed to depolymerize MT in an ATP-dependent manner and regulate MT dynamics (Desai et al., 1999; Trofimova et al., 2018; Ruiz-Reig et al., 2022). In humans, single *de novo* missense mutations in KIF2A were found in patients with early-life epilepsy, posterior pachygyria, microcephaly, and partial agenesis of corpus callosum (Poirier et al., 2013; Tian et al., 2016; Cavallin et al., 2017; Hatano et al., 2021). Analysis of knockout mice revealed that the protein is necessary for neuronal migration, and axonal elongation, branching, and pruning (Homma et al., 2003; Maor-Nof et al., 2013). *Kif2a KO* mice die shortly after birth, preventing studies of the functional consequences of KIF2A deletion (Homma et al., 2003). Recent studies have used conditional knock-out mice (cKO) to uncover the different KIF2A functions in a temporal and tissue specific manner. Early postnatal deletion of KIF2A, using a tamoxifen-inducible approach, causes defects in hippocampal wiring and epileptic seizures, even though the proliferation and migration of granular cells are not affected (Homma et al., 2018). Specific deletion of KIF2A in pallial glutamatergic neurons during development leads to laminar disorganization in the cortex, abnormal neuronal morphology and connectivity and premature neurodegeneration (Ruiz-Reig et al., 2022). KIF2A is also required for adult neurogenesis and tangential migration of neuroblasts in the rostral migratory stream toward olfactory bulbs (Hakanen et al., 2021).

Here, we deleted KIF2A specifically in GABAergic neurons by crossing *Dlx5/6-Cre-eGFP* with *Kif2a^F/F^* mice. We found that *Dlx5/6-Kif2a cKO* mice display hyperactivity and increased susceptibility to epileptic seizures. Using live imaging and immunostaining analyses, we showed that KIF2A loss altered the migration of cortical interneurons during development and their distribution in the adult cortex and hippocampus. Moreover, they formed fewer inhibitory synapses with pyramidal neurons and mutant mice had reduced levels of GABA_A_α1 and GABA_A_α2, indicating a deficit in GABAergic signaling.

## 2. Materials and methods

### 2.1. Mice

All animal procedures were carried out in accordance with European guidelines (2010/63/UE) and approved by the animal ethics committee of the Université catholique de Louvain under agreement 2019/UCL/MD/006. The day of vaginal plug was considered as embryonic day 0.5 (E0.5). We used the following mouse lines: *Kif2a^F/F^* (Hakanen et al., 2021) and *Dlx5/6Cre-eGFP* (Stenman et al., 2003). To produce *Kif2a^FlF^; Dlx5/6Cre-eGFP* (referred to as *Dlx5/6-Kif2a cKO*), we crossed *Dlx5/6Cre-eGFP*; *Kif2a^F/+^* males with *Kif2a^F/+^* females. *Dlx5/6Cre-eGFP*; *Kif2a^+/+^* (*Dlx5/6*-Control) and *Kif2a^F/F^* mice had no phenotype and were undistinguishably considered as controls. Except from behavioral tests that were performed exclusively on males, all experiments were carried out on both males and female without any distinction of the gender.

### 2.2. PTZ-induced epileptic seizures

To evaluate epileptic susceptibility in mice, experiments were performed as described in (Shimada and Yamagata, 2018). PTZ solution (Sigma P6500, 10 mg/mL in sterile 0.9% NaCl) was prepared freshly on the day of use. P40 mice were placed in an observation cage for a 3 min habituation period and then injected with an intraperitoneal single dose of PTZ (dose 35 mg/Kg). Following injection, mice behavior was monitored for 30 min and classified according to following scoring: 0: normal behavior, no abnormality; 1: immobilization, lying on belly; 2: head nodding, facial, forelimb, or hindlimb myoclonus; 3: continuous whole-body myoclonus, myoclonic jerks, tail held up stiffly; 4: rearing, tonic seizure, falling down on its side; 5: tonic–clonic seizure, falling down on its back, wild rushing and jumping; 6: death (Shimada and Yamagata, 2018).

### 2.3. Behavioral analyses

Behavioral experiments were conducted on P40 control and *Dlx5/6-Kif2a cKO* males. The open-field test was used to assess an unforced ambulation as mice can move freely without any influence of the examiner. Briefly, mice were placed in a square arena (60 cm × 60 cm) and video tracked (Ethovision 6.1, Noldus; Wageningen, The Netherlands) for 20 min. The total distance traveled by the animals, the velocity and the time spent in the center vs. periphery were measured (Mignion et al., 2013).

### 2.4. Immunofluorescence and *in situ* hybridization

Embryos were fixed in 4% paraformaldehyde (PFA) in 0.1 M phosphate buffer (PB), pH 7.4, at room temperature (RT) for 2 h. Mice were perfused transcardially with PFA 4%, in 0.1 PBS pH 7.4 or PFA 4%, picric acid 15% in 0.1 M PB for GABA_A_α2 staining. Brains were harvested and post-fixed in the same fixative for 2 h at room temperature (RT) for immunohistochemistry and overnight (ON) at 4°C for *in situ* hybridization (ISH). Embryonic and postnatal brains were washed in PBS, embedded in 4% agarose, and sectioned with a Leica VT1000S vibratome (80 and 40 μm for embryonic and postnatal brains, respectively). Immunohistochemical staining was performed as previously described (Touzot et al., 2016). We used the following primary antibodies: Chicken anti-GFP (Aves-lab GFP-1020, 1:2,000); Rabbit anti-KIF2A (Proteintech 13105-1-AP, 1:500); Rabbit anti-DsRed (Takara biosciences 632496, 1:2,000); Guinea pig anti-TRIM46 (Synaptic Systems 377005, 1:500); Rabbit anti-Parvalbumin (Swant PV25, 1:2,000); Mouse anti-Vgat (Synaptic Systems 131011, 1:1,000); Rabbit anti-GABA_A_α2 (Synaptic Systems 224103, 1:500); Rabbit anti-Calretinin (Swant 7697, 1:2,000); Mouse anti-Reelin (G10, 1:2,000; de Bergeyck et al., 1998). Different AlexaFluor-conjugated secondary antibodies (Invitrogen, 1:800) were used. After immunohistochemistry the sections were incubated with DAPI (Sigma D9564 100 μM) for 5 min and mounted with Mowiol. ISH as described in (Ruiz-Reig et al., 2017). Brain vibratome sections were hybridized with a biotinylated *GAD67* riboprobe from the GAD67 plasmid (van den Berghe et al., 2013). Sections were dehydrated in ethanol, incubated twice in toluene for 10 min, and mounted with Neo-Mount® medium (Merck 109016).

### 2.5. Western blotting

Proteins were isolated from mouse brain cortices and hippocampus as described in Ruiz-Reig et al. (2022). Equal volumes of protein (20 μg) were loaded on 4%–12% Bis-Tris (BoltTM ThermoFhiser NW00080 and NW04125), separated by MOPS running buffer (BoltTM Thermo Fisher B000102), and transferred to PVDF membranes (Millipore ISEQ00005). Membranes were incubated with the following antibodies: Chicken anti-GAPDH (Millipore AB2302, 1:5,000); Rabbit anti-KIF2A (AbCam ab71160, 1:2,000); Mouse anti-Vgat (Synaptic Systems 131011, 1:2,000); Mouse anti-GAD67 (Millipore MAB5406, 1:2,000); Rabbit GABA_A_α1 (Alomone Labs AGA001, 1:1,000); Rabbit GABA_A_α2 (Synaptic Systems 224103, 1:2,000); Rabbit GABA_A_α3 (Alomone Labs AGA003, 1:1,000); GABA_A_α5 (Alomone Labs AGA025, 1:1,000). Membranes were then incubated with HRP-coupled secondary antibodies (1:20,000) and revealed using SuperSignal™ West Pico PLUS Chemiluminescent Substrate (Thermo Fisher 34577). Signals were detected with Fusion Pulse platform (Vilber) and quantified with Fiji (ImageJ). Values were normalized to GAPDH.

### 2.6. Organotypic brain culture and live imaging

Dlx5/6-positive embryos were collected at E13.5 and dissected in ice-cold complete HBSS (cHBSS; HBSS 1x, HEPES 25 mM, D-glucose 30 mM, CaCl_2_ 1 mM, MgSO_4_, NaHCO_3_ 4 mM; Tucker et al., 2006). The embryonic brains were embedded in a solution of 3%–4% low melting point agarose (Fisher Scientific BP165-25) in HBSS, sectioned coronally into 250 μm slices on a Leica VT1000S vibratome, and transferred to poly-L-lysine/laminin-coated transwell membrane inserts (Picmorg50, Millicell®) within a glass-bottomed cell culture dish (627860, Cellview, Greiner bio-one). Each well was filled with prewarmed 1.8 ml of slice culture media (Basal Medium Eagle, cHBSS, D-glucose 20 mM, L-glutamine 1 mM, Penicillin–Streptomycin 100 units/mL and 0.1 mg/mL, respectively). This volume is important to provide the appropriate air-medium interface for the slices. Slices were grown for ~2 h at 37°C prior time lapse. Live imaging was performed with an inverted Zeiss Axio Observer microscope equipped with environmental chamber (37°C, 5% CO2; Pecon) and Zeiss Axiocam 503 mono camera. Time lapses of organotypic brain slices were taken using a 10x (NA = 0.45) objective (Zeiss). Images were captured every 2 min for 8–12 h and each image was generated by compressing 5 z-stack images (4 μm interval) into a single frame. We considered 6 h of time lapse for migration analysis. Migrating neuron tracking was performed manually with ImageJ plugin MtrackJ. For directionality, interneurons trajectories were transferred to begin from center point and directionality was quantified from the trajectory’s endpoints. Stationary neurons (moving less than 15 μm/h) were excluded for velocity and net displacement analyses.

### 2.7. *In utero* electroporation

The plasmids used in this study were: pCag-DsRed (Addgene #11151; 0.7 μg/μL) and pCAG_GPHN.FingR-eGFP-CCR5TC (Addgene #46296; 0.8 μg/μL; Gross et al., 2013). The DNA was purified with a Maxiprep Endofree Kit (Macherey-Nagel, Düren, Germany), and diluted in 1x phosphate-buffered saline (PBS). The DNA solution (with 0.05% Fast green) was injected into the lateral ventricle of E14.5 embryos using pulled glass pipettes. The embryos were electroporated using tweezers-type electrodes (CUY650-5). Five square electric pulses were passed (38 V, 50 ms interval cycle length, 950 ms interval pause).

### 2.8. Data analysis and image processing

Images were captured with a digital camera coupled with an inverted Zeiss Axio Observer microscope or in a Laser Scanning confocal microscope (Olympus Fluoview FV1000). Figures were prepared using Adobe Photoshop and Adobe Illustrator CC 2019, and 2D mosaic reconstructions were produced when needed using the Photomerge tool of Photoshop software package. Cell counting was conducted manually using Fiji software (ImageJ). For gephyrin (GPHN) cluster analysis on electroporated neurons and GABA_A_α2+/Vgat+ clusters in the hippocampus, confocal images were obtained with a 63x objective and 3x digital zoom. To identify the AIS of electroporated neurons, sections were immunostained with TRIM46 antibody and images were taken along the TRIM46 positive region. Image stacks (0.5 μm step size) were reconstructed and analyzed with Fiji software to calculate GPHN+ cluster density in dendrites and AIS, and GABA_A_α2+/Vgat+ cluster density in the pyramidal layer of the hippocampus. Cell quantification data and graphs were constructed using Prism 9 (GraphPad, San Diego, CA, United States) software. A minimum of 3 animals per genotype was used for all analyses and quantifications. The exact sample size is specified in the figure legends. For each genotype, a mean value was calculated from all counted sections. Error bars represent the standard error of the mean (SEM). We performed a Shapiro Wilks test to evaluate the distribution of the data. We then used two-tailed Student’s *t*-test when the data followed a normal distribution and Mann–Whitney test when it did not (n.s. not significant, *p < 0.05, **p < 0.01, ***p < 0.001). Observations without quantification have been validated and successfully reproduced in a minimum of three different animals.

## 3. Results

### 3.1. Conditional deletion of KIF2A in cortical interneurons triggers hyperactivity and epileptic seizures

**Figure 1:**
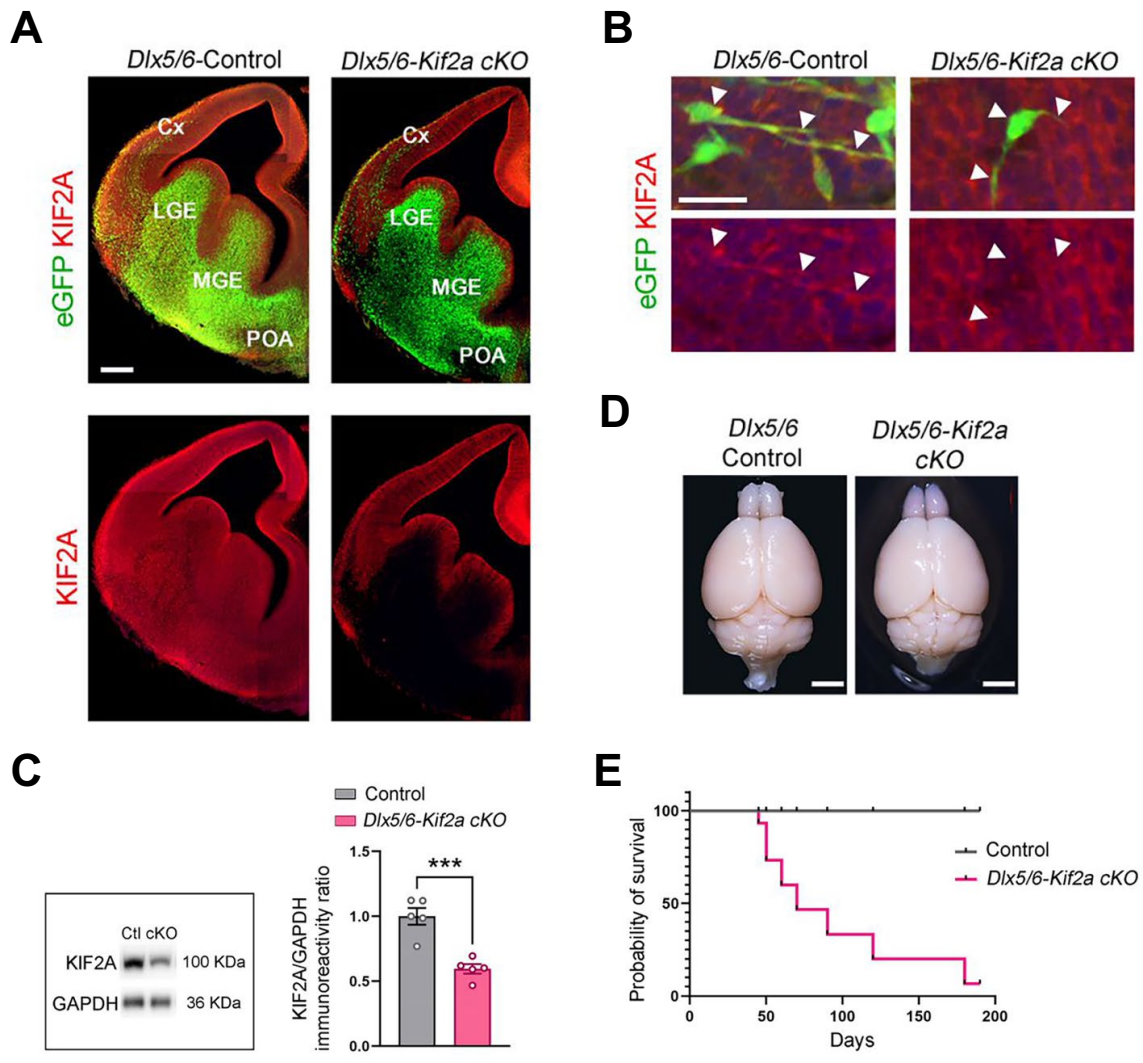
Conditional deletion of KIF2A in the mouse forebrain GABAergic neurons results in short survival. **(A)** Coronal sections of control and *Dlx5/6-Kif2a cKO* embryos showing the expression of Dlx5/6-Cre-eGFP (green) and KIF2A (red) at embryonic day 13.5 (E13.5). **(B)** Representative images of migrating neuron in control and *Dlx5/6-Kif2a cKO* embryos. White arrowheads delineate neuronal shape. **(C)** Western blot analysis and quantification of the relative amount of KIF2A from control (Ctl) and *Dlx5/6-Kif2a cKO* (cKO) adult telencephalon (*n* = 5 mice per genotype). **(D)** Representative images of whole brains of the indicated genotypes at P40. **(E)** Kaplan–Meier curve depicting a decrease in survival of *Dlx5/6-Kif2a cKO* as compared to control mice (*n* = 9 mice per genotype). Cx, cortex; LGE, lateral ganglionic eminence; MGE, medial ganglionic eminence; POA, preoptic area. Scale bars **(A)** 200 μm, **(B)** 25 μm, **(D)** 2 mm. Data are represented as mean ± SEM. Values were obtained by unpaired Student’s t-test; *p < 0.05, **p < 0.01, and ***p < 0.001.

**Figure 2:**
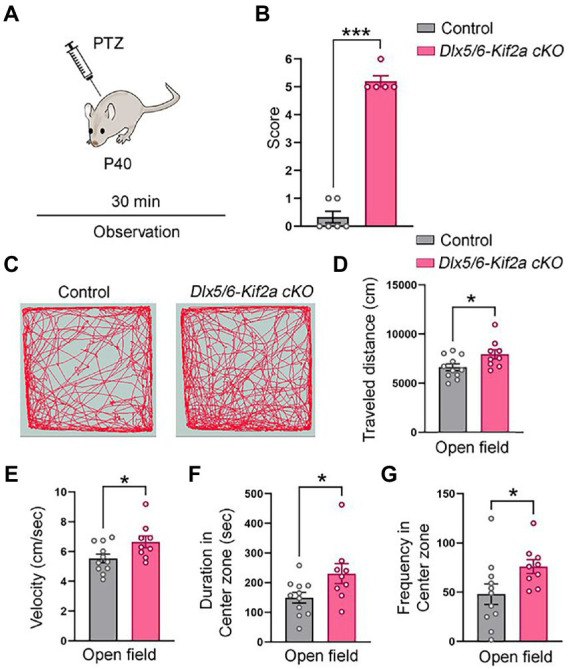
*Dlx5/6-Kif2a cKO* mice are hyperactive and more susceptible to epilepsy. **(A)** Experimental design of PTZ-induced seizure susceptibility experiments. **(B)** Graph showing mean score of seizure behavior for each genotype during the 30 min post-injection period (control mice *n* = 6, *Dlx5/6-Kif2a cKO* mice n = 5). **(C)** Illustrative example of mice movement in open field test. **(D–G)** Graphs showing the traveled distance **(D)** (control: 6630 ± 344 cm, *n* = 11; *Dlx5/6-Kif2a cKO*: 7951 ± 472 cm, *n* = 9), velocity **(E)** (control: 5.54 ± 0.29 cm/s, *n* = 11; *Dlx5/6-Kif2a cKO*: 6.65 ± 0.4 cm/s, *n* = 9), time in the center zone **(F)** (control: 149.7 ± 18 s, *n* = 11; *Dlx5/6-Kif2a cKO*: 230.5 ± 33.8 s, *n* = 9) and frequency in the open field center **(G)** (control: 48.04 ± 10.35, *n* = 11; *Dlx5/6-Kif2a cKO*: 76 ± 7.02, *n* = 9). Data are represented as mean ± SEM. Values were obtained by unpaired Student’s t-test; *p < 0.05, **p < 0.01, and ***p < 0.001.

During embryonic development, KIF2A is ubiquitously expressed in the forebrain (Figure 1A). We inactivated the *Kif2a* gene in GABAergic neurons by crossing the *Dlx5/6-Cre-IRES-eGFP* line with *Kif2a^F/F^* mice (Stenman et al., 2003; Hakanen et al., 2021). We used the *eGFP* transgene to monitor expression of Dlx5/6 promoter, and KIFA2 immunofluorescence to monitor recombination (Figure 1A). In *Dlx5/6-*Control animals (i.e., *Dlx5/6Cre-eGFP;Kif2a^+/+^*), eGFP-positive cells were found in the ganglionic eminences, preoptic area (POA), and cortical interneurons migrating tangentially toward the cortex (Figures 1A,B). In *Dlx5/6-Kif2a cKO* (i.e., *Dlx5/6Cre-eGFP;Kif2a^F/F^*), KIF2A expression was lost specifically in Dlx5/6-Cre-eGFP positive neurons (Figures 1A,B). Western blot analysis confirmed a reduction of KIF2A protein level in the mutant adult telencephalon (−40 ± 7%, *p* = 0.0006; Figure 1C; Supplementary Figure S1A). We did not detect any difference in the brain size between control and mutant mice (Figure 1D). However, *Dlx5/6-Kif2a cKO* mice had a shorter survival compared with controls (Figure 1E) and displayed spontaneous epileptic seizures. To investigate this further, we injected Pentylenetetrazol (PTZ), a GABA_A_ receptor antagonist, and scrutinized mice behavior for 30 min (Figure 2A). After a single PTZ injection, four (out of five) *Dlx5/6-Kif2a cKO* mice had tonic–clonic seizure (score 5), and one died (score 6), while control mice had a score of 0 or 1 (immobilization; Figure 2B; Supplementary Movie S1). In addition, *Dlx5/6-Kif2a cKO* mice were hyperactive; they moved more (+19.9 ± 8.62%, *p* = 0.033; Figures 2C,D) and faster (+20 ± 8.67%, *p* = 0.033; Figures 2C,E) than controls in the open field test. Moreover, they spent more time in the center of the open field (duration: +54 ± 24.3%, *p* = 0.039 and frequency: +58.2 ± 27.3%; *p* = 0.047; Figures 2F,G). These results indicate that deletion of KIF2A in cortical GABAergic neurons triggers hyperactivity and increased susceptibility to epileptic seizures.

### 3.2. KIF2A is necessary for tangential migration of cortical interneurons

**Figure 3:**
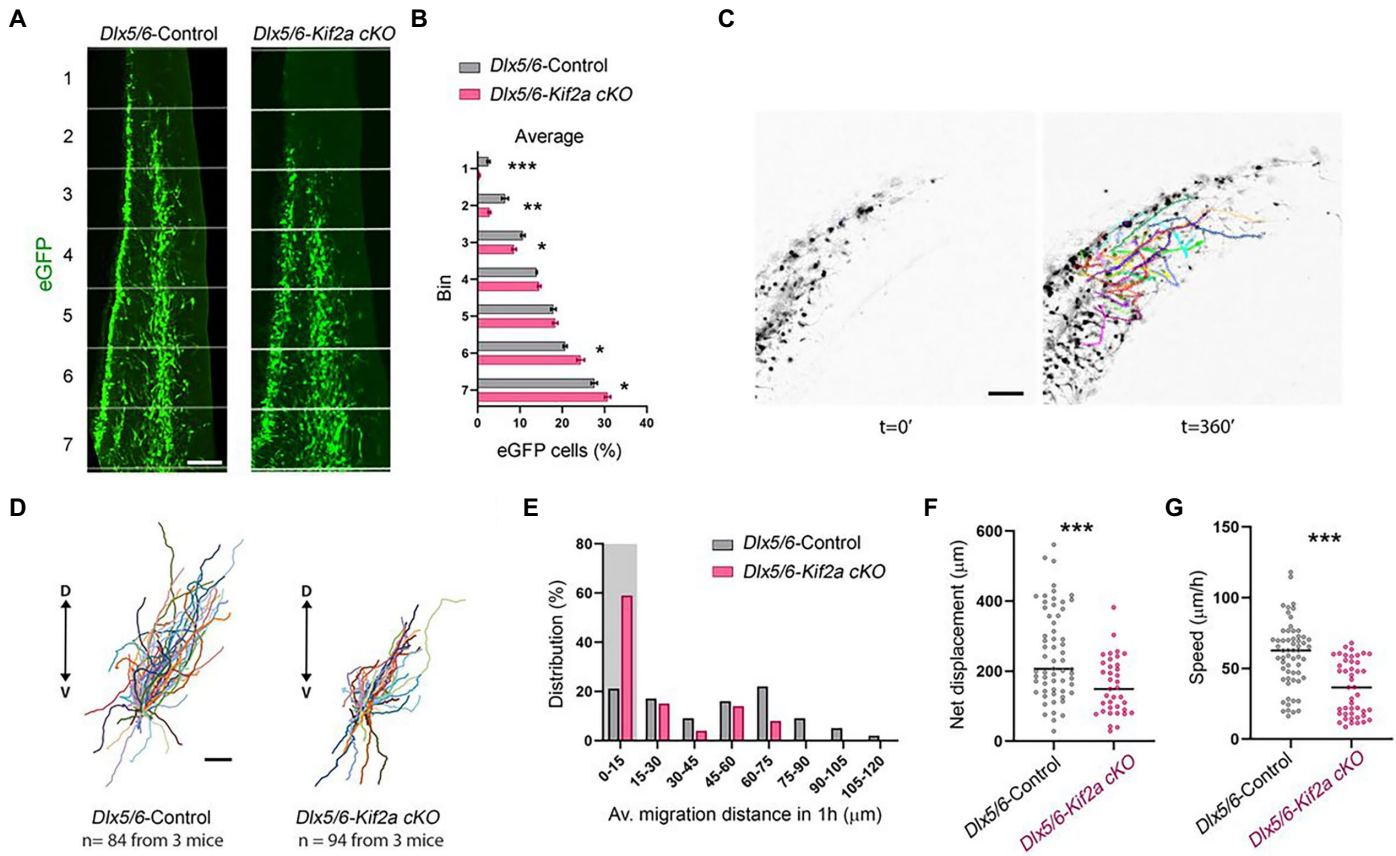
Cell-autonomous requirement of KIF2A for interneuron migration. **(A)** Distribution of Dlx5/6-Cre-eGFP-positive interneurons in 7 equal bins along the migratory pathway at E13.5. **(B)** Percentage of cells in each bin for control and *Dlx5/6-Kif2a cKO* mice (control *n* = 4 and *Dlx5/6-Kif2a cKO n* = 6 embryos). **(C)** Representative time lapse sequence illustrating tracking of interneurons in control and *Dlx5/6-Kif2a cKO* mice. **(D)** Schematic illustration of migratory trajectories starting from the center. **(E)** Distribution of interneurons by migrated distance in control and *Dlx5/6-Kif2a cKO* mice (control *n* = 81, *Dlx5/6-Kif2a cKO n* = 92 from 3 different embryos each genotype). The shaded area depicts stationary interneurons (0–15 μm), which were removed from further analysis. **(F)** Net displacement in control and *Dlx5/6-Kif2a cKO* mice (control: 251 ± 16.4 μm, *n* = 61; *Dlx5/6-Kif2a cKO*: 157.9 ± 13.4 μm *n* = 34 from 3 different embryos each genotype). **(G)** Speed in control and *Dlx5/6-Kif2a cKO* mice (control: 59.37 ± 3 μm, *n* = 60; *Dlx5/6-Kif2a cKO*: 36.22 ± 2.9 μm *n* = 45 from 3 different embryos each genotype). Scale bars **(A,C,D)** 100 μm. Data are represented as mean ± SEM. Values were obtained by unpaired Student’s t-test **(B)** or Mann-Whitney test **(F,G)**; *p < 0.05, **p < 0.01, and ***p < 0.001.

We next aimed to investigate the relationship between KIF2A deficiency, development of cortical interneurons, and abnormal behavior of mutant mice. We analyzed the distribution of eGFP-positive neurons in control and *Dlx5/6-Kif2a cKO* animals during development. At embryonic day 13.5 (E13.5), GABAergic interneurons invaded the cerebral cortex through two main migratory streams (Figure 3A). We subdivided the cortex into seven equal bins and quantified the number of migrating interneurons in each bin. The percentage of eGFP-positive cells reaching the most dorsal bins was markedly reduced *Dlx5/6-Kif2a cKO* (Figure 3B). Accordingly, more eGFP-positive cells accumulated in the ventral bins (Figure 3B), indicating a delayed tangential migration. These differences were not region dependent as the same delay was detected at different rostrocaudal levels (Supplementary Figure S2). We analyzed the migratory behavior of cortical interneurons using live imaging (Supplementary Movie S2). We assessed different parameters in acute brain slices by tracking migrating cells at mid-cortical positions for a period of 6 h (Figure 3C; Supplementary Movies S3, S4). Several *Dlx5/6-Kif2a cKO* cells lost directionality and migrated ventrally (Figure 3D). We segregated the cells into bins depending on the traveled distance (Figure 3E) and considered those which moved less than 15 μm in 1 h as stationary cells. In mutant animals, 59% of the eGFP-positive cells were stationary vs. 21% in control animals (Figure 3E). The maximum migratory length was 118 μm for control neurons vs. 67.9 μm in mutant embryos (Figure 3E). We excluded stationary cells and evaluated the net displacement and velocity. Both were reduced in *Dlx5/6-Kif2a cKO* embryos (net displacement: −37.1 ± 9.37%, *p* = 0.0004; velocity: −39 ± 7.22%, *p* < 0.0001; Figures 3F,G), suggesting that KIF2A regulates the tangential migration of the cortical interneurons in a cell-autonomous manner.

### 3.3. KIF2A deletion alters the distribution of GABAergic neurons in the cortex and hippocampus

**Figure 4:**
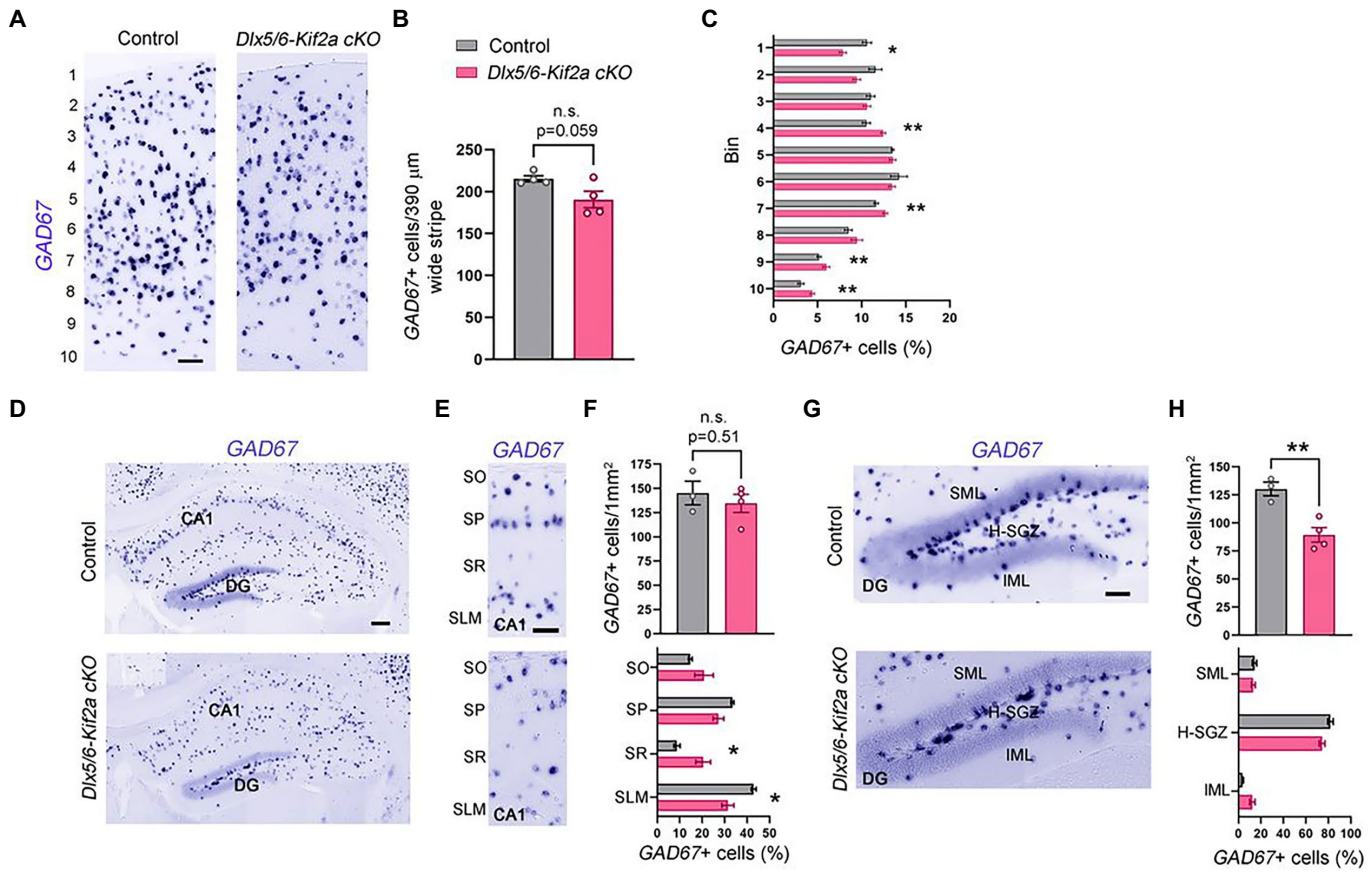
KIF2A deficiency alters the distribution of GABAergic neurons in the cortex and hippocampus. **(A)** Coronal sections at the level of the somatosensory cortex hybridized with *GAD67* riboprobe in control and *Dlx5/6-Kif2a cKO* mice at P40. **(B)** Mean value of *GAD67*-positive neurons in 390 μm wide stripe (control: 215.3 ± 3.7, *n* = 4; *Dlx5/6-Kif2a cKO*: 190.5 ± 10, *n* = 4). **(C)** Percentage of *GAD67*-positive neurons in each bin in control and *Dlx5/6-Kif2a cKO* mice. **(D)** Hippocampal Coronal sections hybridized with *GAD67* riboprobe in control and *Dlx5/6-Kif2a cKO* mice. **(E)** Magnification of the CA1 region depicting *GAD67*-positivecells. **(F)** Upper panel, mean density of *GAD67*-positive neurons in CA1 (control: 145.2 ± 12.2 cells/1 mm^2^, *n* = 3; *Dlx5/6-Kif2a cKO*: 134.5 ± 9.4 cells/1 mm^2^ *n* = 4). Lower panel, distribution of *GAD67*-positive neurons (percentage of *GAD67*-positive cells per subregion in *GAD67*-positive cells in CA1 of the corresponding genotype). **(G)** Magnification of the dentate gyrus (DG) highlighting *GAD67-*positive cells. **(H)** Upper panel, mean density of *GAD67*-positive neurons in DG (control: 130.2 ± 6.2 cells/1mm^2^, *n* = 3; *Dlx5/6-Kif2a cKO*: 89.36 ± 6.6 cells/1mm^2^ *n* = 4). Lower panel, distribution of *GAD67*-positive neurons per DG layer and per genotype. DG, dentate gyrus; SO, stratum oriens; SP, stratum pyramidale; SR, stratum radiatum; SLM, stratum lacunosum-moleculare; SML, superior molecular layer; IML, inferior molecular layer; H-SVZ, hilus-subventricular zone. Scale bars **(A,E,G)** 100 μm, **(D)** 200 μm. Data are represented as mean ± SEM. Values were obtained by unpaired Student’s t-test; *p < 0.05, **p < 0.01, and ***p < 0.001.

To analyze whether defects in neuronal migration during embryogenesis affect the total number of cortical interneurons in the mature cortex and hippocampus, we evaluated the number of GABAergic neurons at P40 using *in situ* hybridization for *GAD67*. The density of *GAD67+* cells in the somatosensory cortex (S1) was reduced in mutant mice, but the difference was not statistically significant (−11.5 ± 4.96%, *p* = 0.059; Figures 4A,B). To analyze the distribution of cortical interneurons, we subdivided S1 into 10 equal bins and calculated the percentage of *GAD67+* cells per bin. We found more GABAergic neurons in the lower bins (layers V–VI) and less in upper bins (layers I–II) in mutant mice compared with control littermates (Figure 4C). To test whether alterations in distribution are related to specific interneuron populations, we analyzed the density and distribution of three cortical interneuron subtypes, namely, parvalbumin (PV+), calretinin (CR+) and reelin (RLN+) expressing interneurons (Supplementary Figure S3). The density of these populations was similar between the two genotypes. However, the distribution of CR+ and RLN+ was significantly affected. There were more CR+ and RLN+ cells in bin 7, 4, respectively; and less RLN+ in bin 1 (Supplementary Figures S3B,E,F). We also analyzed the density and distribution of GABAergic neurons in the hippocampus (Figure 4D). There was no significant difference in the density of these cells in the hippocampal CA1 region (Figures 4E,F). However, *GAD67+* cells were more randomly distributed along the different layers of mutants, contrasting with a clear concentration of these cells in the Stratum pyramidale (SP) and the Stratum Lacunosum Molecular (SLM) in controls (Figures 4E,F). The density of GAD67+ cells was significantly reduced in the dentate gyrus (−31.44 ± 7.14%, *p* = 0.007; Figures 4G,H). In both genotypes, the majority of the *GAD67+* cells were localized in the hilus-subgranular zone (H-SGZ; Figures 4G,H). Overall, these results indicate that KIF2A is necessary for the correct distribution of GABAergic neurons in the cortex and hippocampus.

### 3.4. GABAergic function is impaired in *Dlx5/6-Kif2a cKO* mice

**Figure 5:**
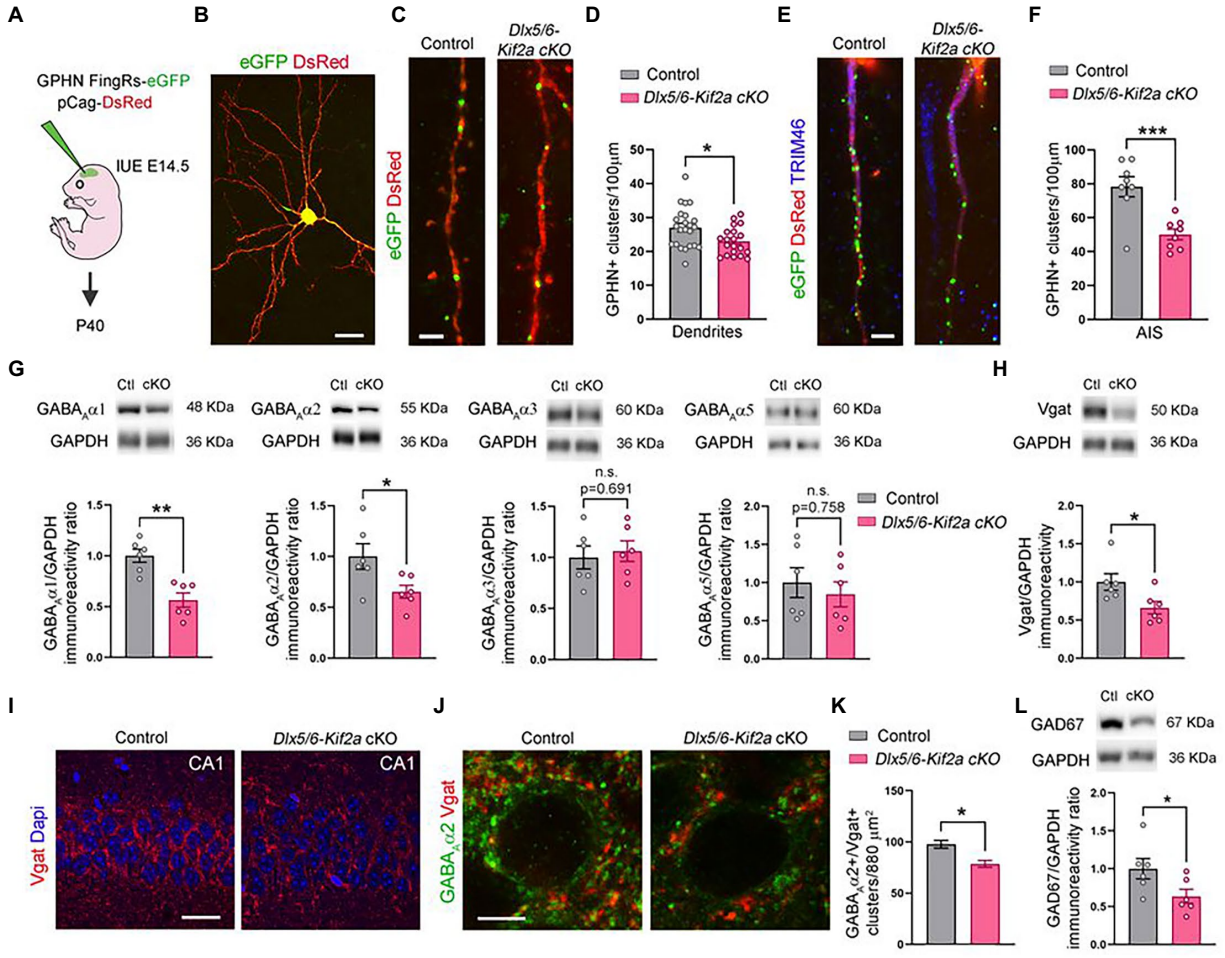
Deletion of KIF2A affects inhibitory signaling in cortex and hippocampus. **(A)** Experimental design of *in utero* (IUE) experiments. **(B)** Control pyramidal neuron electroporated at E14.5 with DsRed and gephyrin (GPHN) FingRs-eGFP plasmids and analyzed at P40. **(C)** High magnification of dendrites showing gephyrin-positive clusters (eGFP signal) in the indicated genotype. **(D)** Quantification of gephyrin positive (GPHN+) clusters density in dendrites (control: 27.02 ± 1.23 clusters/100 μm, *n* = 23 from 3 mice; *Dlx5/6-Kif2a cKO*: 22.99 ± 0.92 cluster/100 μm *n* = 20 from 2 mice). **(E)** Axon initial segment (AIS) showing gephyrin-positive clusters (eGFP) in indicated genotypes. **(F)** Quantification of gephyrin positive (GPHN+) clusters density in the AIS (control: 78.28 ± 5.94 cluster/100 μm, *n* = 8 from 3 mice; *Dlx5/6-Kif2a cKO*: 50.15 ± 3.14 cluster/100 μm *n* = 8 from 2 mice). **(G)** Western blot analysis and quantification of the relative amount of different GABA_A_ receptor subunits in P40 cortical and hippocampal extracts of control (Ctl) and *Dlx5/6-Kif2a cKO* (cKO) mice (*n* = 6 for each genotype). **(H)** Western blot analysis and quantification of the relative amount of Vgat in P40 cortical and hippocampal extracts (n = 6 for each genotype). **(I,J)** Immunofluorescence for Vgat **(I)** and GABAAα2 and Vgat **(J)** in the CA1 pyramidal layer at P40. **(K)** Quantification of GABA_A_α2+/Vgat+ cluster density in the CA1 pyramidal layer of hippocampus (control: 97.78 ± 3.82, *n* = 3 mice; *Dlx5/6-Kif2a cKO*: 78.61 ± 3.48, *n* = 3 mice). **(L)** Western blot analysis and quantification of the relative amount of GAD67 in P40 cortical and hippocampal extracts (*n* = 6 for each genotype). Scale bars **(B)** 20 μm, **(C,E)** 2.5 μm, **(I)** 25 μm, **(J)** 5 μm. Data are represented as mean ± SEM. Values were obtained by unpaired Student’s t-test; *p < 0.05, **p < 0.01, and ***p < 0.001.

Given that ablation of KIF2A from excitatory neurons affects the number of glutamatergic synapses (Ruiz-Reig et al., 2022), we evaluated the impact its deletion on GABAergic synapse formation in *Dlx5/6-Kif2a cKO*. We analyzed the density of the inhibitory postsynaptic protein gephyrin. We electroporated a plasmid expressing FingRs fused with eGFP at E15.5 to label active gephyrin positive clusters (GPHN-eGFP) on pyramidal neurons (Gross et al., 2013; Figures 5A,B). At P40, pyramidal neurons in the somatosensory cortex displayed a reduced density of gephyrin signal (GPHN-eGFP) in dendrites (−14.9 ± 5.8%, *p* = 0.014; Figures 5C,D), and more noticeably, in the axon initial segment (AIS) highlighted by TIMP46 immunostaining (van Beuningen et al., 2015; −35.9 ± 8.6%, *p* = 0.0009; Figures 5E,F). GABA_A_ receptors (GABA_A_Rs) mediate a large majority of fast-inhibitory synapses in the adult brain. We used Western blot analysis to assess the relative protein levels of the most abundant subunits of GABA_A_ receptors in cortex and hippocampus. Mutant mice exhibited a significant reduction of GABA_A_α1 (−43.5 ± 9.5%, *p* = 0.001) and GABA_A_α2 (−34.7 ± 14.2%, *p* = 0.035) subunits, while GABA_A_α3 and GABA_A_α5 were unaffected (Figure 5G; Supplementary Figure S1B). The levels of vesicular GABA transporter (Vgat) were also considerably reduced in mutant mice (−33.7 ± 13.6%, *p* = 0.033; Figure 5H; Supplementary Figure S1C). To corroborate the reduction of inhibitory synapses in the mutant hippocampus, we used immunofluorescence for Vgat and GABA_A_α2, and noticed a significant reduction in Vgat staining in the pyramidal layer of CA1 along with a reduction in GABA_A_α2+/Vgat+ cluster density (−19.6% ± 5.2%, *p* = 0.021; Figures 5I–K). To determine whether changes in inhibitory synapses result from a change in the number of fast-spiking interneurons, we analyzed the density of parvalbumin-positive neurons in the CA1 region and did not find a significant difference between control and mutant mice (Supplementary Figure S4). Western blot analysis from cortical and hippocampal lysates, revealed a significant reduction of GAD67 protein levels in mutant mice (−36.4 ± 16.3%, *p* = 0.049; Figure 5L; Supplementary Figure S1C). Since GAD67 catalyzes the conversion of glutamate into GABA, reduced GAD67 levels suggest an impairment in GABA production in mutant animals. These results indicate that KIF2A is necessary for the development and/or maintenance of inhibitory synapses in the cortex and hippocampus and GABAergic signaling.

## 4. Discussion

KIF2A is a MT depolymerizing protein with important roles in cerebral development and function. Here, we report that KIF2A is essential for tangential migration of cortical interneurons during development and for the formation and/or maintenance of inhibitory synapses in the adult brain. Deletion of KIF2A in cortical interneurons disrupts their migration, reduces the number inhibitory synapses, and compromises the balance between excitation and inhibition leading to epilepsy.

The cytoskeleton is involved in many cellular processes, such as proliferation of neural progenitors as well as neuronal polarization, migration, wiring, and plasticity. Therefore, mutations in genes coding for cytoskeleton-related proteins are often associated with disorders of neuronal migration in the cortex, including subcortical band heterotopias (SBH), lissencephaly and interneuropathies among others (Stouffer et al., 2016). Failure in glutamatergic neurons positioning results their accumulation in the white matter, abnormal layering, or folding of the cerebral cortex (lissencephaly, pachygyria, polymicrogyria). In humans, most cases of lissencephaly are caused by mutations in *LIS1* and *DCX* genes, both of which code for proteins implicated in MT function. LIS1 is essential to nucleus-centrosome coupling during migration while DCX stabilizes MT. Given that *LIS1* and *DCX* genes are implicated in radial and tangential migration in the cortex (Reiner, 2013), the origin of epilepsy condition in humans with mutations in these genes remains unclear. Impaired migration, development or function of interneurons, have been associated with epileptic encephalopathies (Katsarou et al., 2017). Examples of genes causing interneuronopathy, and directly associated with aberrant interneuron migration and epilepsy, include the X-linked aristaless-related homeobox (ARX). Patients with *ARX* gene variants suffer intellectual disability and early-life epilepsy. In mice, *Arx* is expressed in the ventricular zone of the pallium and subventricular zone and mantle of the ganglionic eminence (subpallium; Kitamura et al., 2002). Mice with conditional deletion of *Arx* in the ganglionic eminence (*Dlx5/6-Cre-IRES-eGFP;Arx^F/−^*) exhibit interneuron migration abnormalities and epileptic seizures, while mice with targeted deletion in the pallium (*Emx1-Cre;Arx^F/−^*) have no epileptic phenotype (Marsh et al., 2009; Simonet et al., 2015; Marsh et al., 2016).

The role of KIF2A in the radial migration of glutamatergic neurons in the cortex and hippocampus is well documented (Homma et al., 2003; Broix et al., 2018; Gilet et al., 2020; Akkaya et al., 2021; Ruiz-Reig et al., 2022). In the present study, we assessed its function in tangential migration of cortical GABAergic interneurons. We crossed the transgenic line *Dlx5/6-Cre-IRES-eGFP* with the *Kif2a^F/F^* (Stenman et al., 2003; Hakanen et al., 2021) and found that loss of KIF2A affects the velocity, net displacement, and directionality of migration, a phenotype that is similar to that of KIF2-deficient neuroblasts migrating along the rostral migratory stream in the postnatal brain (Hakanen et al., 2021). Despite the abnormal migration during development, the density of GABAergic neurons in the cortex and the CA1 of the hippocampus were not significantly affected in adult mice. Given that 20%–30% of cortical interneurons undergo cell death in the two first postnatal weeks (Wong and Marin, 2019), the final number of cortical interneurons could have been compensated by a reduction in the programmed cell death in *Dlx5/6-Kif2A cKO* mice. Because KIF2A is important for formation and maintenance of excitatory synapses (Ruiz-Reig et al., 2022), we examined inhibitory inputs and found that the loss of KIF2A in GABAergic interneurons reduced the number of active inhibitory synapses, particularly in the AIS of pyramidal neurons. In hippocampus, the protein levels of GABA_A_α1 and GABA_A_α2 were decreased. Importantly, these subunits are enriched around the soma and AIS of pyramidal cells receiving inhibitory synapses from basket and chandelier cells, two main classes of parvalbumin-positive fast-spiking interneurons. Basket cell dysfunction is linked to epilepsy in humans (Jiang et al., 2016), and decreased levels of GABA_A_α1 and GABA_A_α2 are observed in patients with temporal lobe epilepsy and Dravet syndrome, respectively, (Galanopoulou, 2008). We also found a reduction of GAD67 and Vgat protein levels in *Dlx5/6-Kif2a cKO*, suggesting alterations in the production and/or transport of GABA into synaptic vesicles.

Patients with missense mutations in *KIF2A* exhibit so called cortical dysplasia complex with other brain malformations (CDCBM), a type of MCD characterized by posterior agyria/pachygyria, microcephaly and severe motor dysfunction; and most of them suffer childhood epilepsy (Poirier et al., 2013; Tian et al., 2016; Cavallin et al., 2017; Costain et al., 2019; Hatano et al., 2021). Pathogenic variants of KIF2A alter the subcellular localization of KIF2A, thus affecting its ability to bind MT (Poirier et al., 2013; Broix et al., 2018; Gilet et al., 2020). Overexpression of two different KIF2A mutant variants by *in utero* electroporation in the cortex impairs the radial migration of cortical neurons (Broix et al., 2018). It also affects the speed and direction of cortical interneuron tangential migration in organotypic cultures (Broix et al., 2018). Knock-in mice with ubiquitous expression of the mutant variant c.961C > G/p.His321Asp (*Rosa26-Cre; KIF2A^H321D/+^*) have laminar defects in the cortex and hippocampus and are more susceptible to induced epilepsy (Gilet et al., 2020). However, the variant was expressed in both glutamatergic and GABAergic neurons, which precluded the identification of epileptic susceptibility origin. Conditional deletion of KIF2A at the third postnatal week, using a tamoxifen-inducible mouse (*CAGG-Cre-ERTM;Kif2a^F/F^*), also triggers epilepsy (Homma et al., 2018). In these mice, the proliferation, migration, and lamination are preserved, and epilepsy was accredited to abnormal wiring and polarization of granular cells, and dysfunctional excitatory circuits. It would be interesting to investigate if postnatal deletion of KIF2A only in cortical interneurons could affect the maturation and connectivity of these neurons and contribute to the pathophysiological mechanism underlying epilepsy in humans.

## Data availability statement

The original contributions presented in the study are included in the article/Supplementary material, further inquiries can be directed to the corresponding authors.

## Ethics statement

The animal study was reviewed and approved by Experimental Animal Ethics Committee, Université catholique de Louvain (agreement number 2019/UCL/MD/006).

## Author contributions

NR-R, DG-S, and OS performed the experiments. NR-R, PG, and FT designed the research. NR-R and FT wrote the manuscript. All authors contributed to the article and approved the submitted version.

## Funding

This work was supported by the following grants: FNRS PDR T0236.20, FNRS-FWO EOS 30913351, Fondation Médicale Reine Elisabeth, and Fondation JED-Belgique. NR-R and DG-S are Postdoctoral Researcher and Research Fellow at the Belgian Fund for Scientific Research (FNRS), respectively. FT is an Honorary Research Director FNRS.

## Conflict of interest

The authors declare that the research was conducted in the absence of any commercial or financial relationships that could be construed as a potential conflict of interest.

## Publisher’s note

All claims expressed in this article are solely those of the authors and do not necessarily represent those of their affiliated organizations, or those of the publisher, the editors and the reviewers. Any product that may be evaluated in this article, or claim that may be made by its manufacturer, is not guaranteed or endorsed by the publisher.
