# Supplementary material for: Inhibitory synapse dysfunction and epileptic susceptibility associated with KIF2A deletion in cortical interneurons
Source: Front Mol Neurosci. 2023 Jan 17;15:1110986. doi: 10.3389/fnmol.2022.1110986 (PMC9887042; doi:10.3389/fnmol.2022.1110986)
Supplement: Supplementary file 5 [file Data_Sheet_1.pdf]

Supplementary Material

Cortical interneuron dysfunction and epileptic susceptibility associated with KIF2A deletion

Nuria Ruiz-Reig [1]*, Dario Garcia-Sanchez[1], Olivier Shackman[1], Phillipe Gailly[1], Fadel Tissir[1,2]*.

[1] Université catholique de Louvain, Institute of Neuroscience, Brussels, Belgium.

[2] College of Health and Life Sciences, Hamad Bin Khalifa University, Doha, Qatar

* **Correspondence:** Nuria Ruiz-Reig nuria.ruizreig@uclouvain.be or Fadel Tissir ftissir@hbku.edu.qa and fadel.tissir@uclouvain.be

1 Supplementary Figures

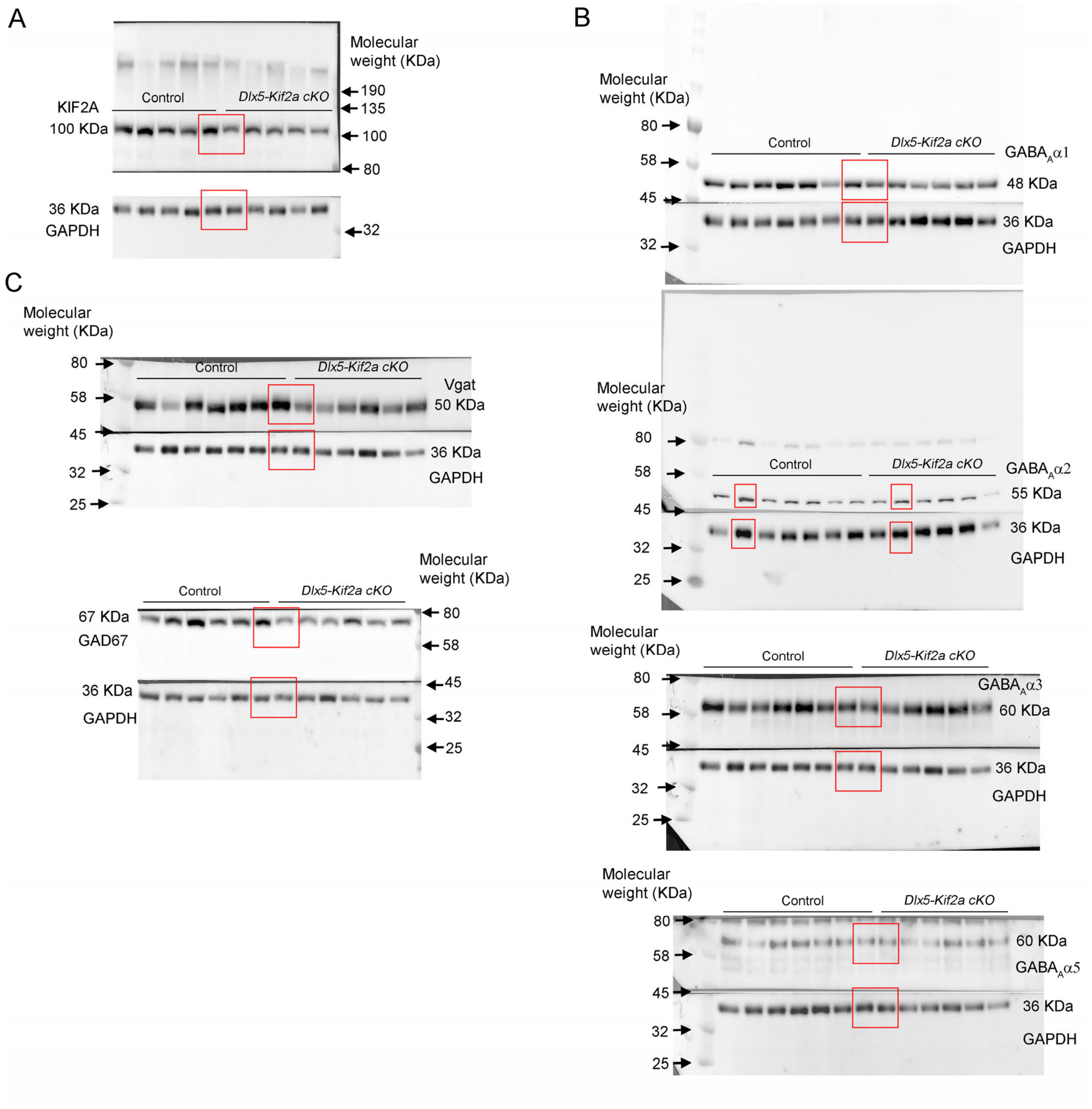

Figure S1. Complete Western blot membranes. Complete membranes of the western blot for Figures 1 and 5. (A) Immunoblot for KIF2A from control and *Dlx5/6-Kif2a cKO* whole telencephalon extracts at adult stages. The boxed was cropped and shown in Figure 1C. (B) Immunoblot for different $GABA_A$ receptor subunits in P40 cortical and hippocampal extracts from control and *Dlx5/6-Kif2a cKO* mice. Boxed Areas were cropped and used as illustration in Figure 5G (C) Immunoblot for Vgat and GAD67 in P40 cortical and hippocampal extracts from control and *Dlx5/6-Kif2a cKO* mice. Boxed areas were cropped and shown in Figure 5H and 5L, respectively.

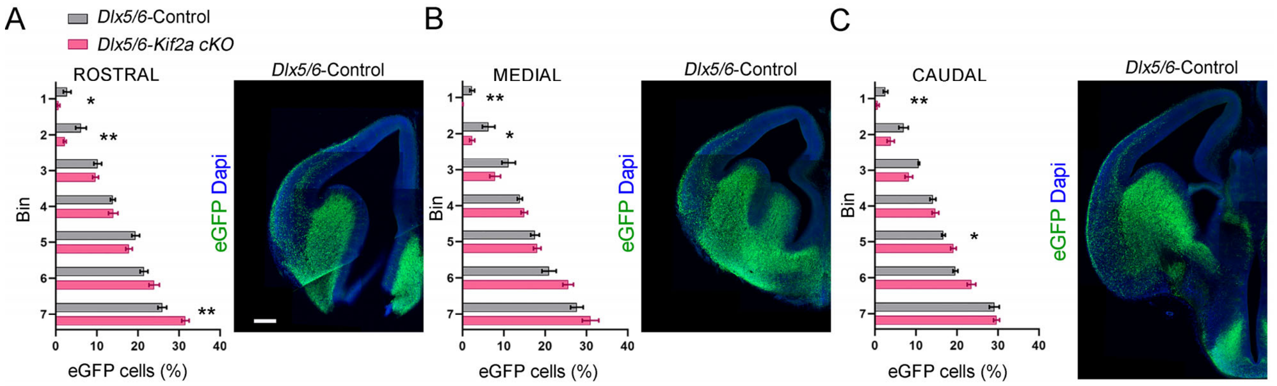

Figure S2. Dlx5/6-positive interneuron migration defects in *Dlx5/6-Kif2a cKO* mice are not region dependent. (A-C) Interneuron distribution in the cortex and immunofluorescence for Dlx5/6-eGFP in coronal sections at three different rostro-caudal levels at E13.5; rostral (A), medial (B) and caudal (C). Scale bar (A) 200 µm. Data are represented as mean ± SEM. Values were obtained by unpaired Student's t-test; *P < 0.05, **P < 0.01, and ***P < 0.001.

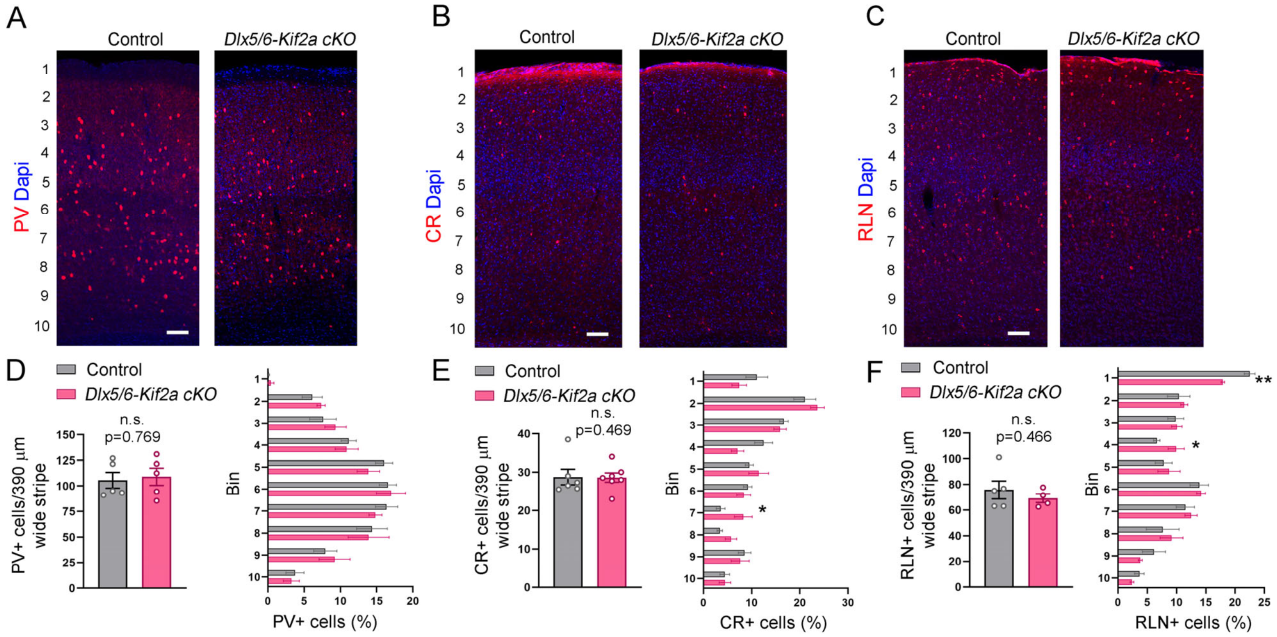

Figure S3. Cortical interneuron density and distribution in control and *Dlx5/6-Kif2a* mice. (A-C) Immunofluorescence in coronal section at the levels of the somatosensory cortex labeling PV (A), CR (B) and RLN positive interneurons in P40 control and *Dlx5/6-Kif2a cKO* mice. (D-F) Quantification of the density and distribution of PV (D) (density; control: 105.6 ± 7.7; *Dlx5/6-Kif2a cKO*: 109 ± 8.4, n=5 mice per genotype), CR (E) (density; control: 29.4 ± 2.3, n=5 mice; *Dlx5/6-Kif2a cKO*: 28.37 ± 1.4, n=6 mice) and RLN (F) (density; control: 76.17 ± 5.5; *Dlx5/6-Kif2a cKO*: 68.5 ± 3.5, n=4 mice per genotype) interneurons in 390 µm wide stripe. PV, parvalbumin; CR, calretinin; RLN, reelin. Scale bar (A-C) 100 µm. Data are represented as mean ± SEM. Values were obtained by unpaired Student's t-test (A and C) or Mann-Whitney test (B); *P < 0.05, **P < 0.01, and ***P < 0.001.

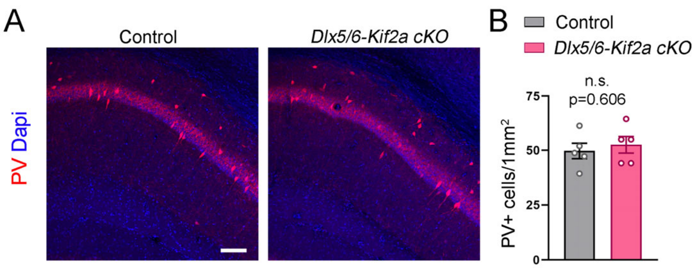

Figure S4. KIF2A deletion does not affect parvalbumin (PV) positive interneuron density in the hippocampus. (A) Immunofluorescence for PV in coronal sections at the level of the CA1 region of control and *Dlx5/6-Kif2a cKO* mice at P40. (B) Quantification of PV density in the CA1 region (control: 49.74 ± 3.58 cells/1mm^2, n=5 mice; *Dlx5/6-Kif2a cKO*: 52.57 ± 3.88 cells/1mm^2, n=5 mice). Scale bar (A) 100 µm. Data is represented as mean ± SEM.

2 Supplementary Movies

Movie S1. PTZ-induced seizure in *Dlx5/6-Kif2a cKO* mice. Monitoring of control and *Dlx/65-Kif2a cKO* mice during the PTZ-post injection period. The *Dlx5/6-Kif2a cKO* mouse exhibits a tonic-clonic seizure (score 5; falling down on the back and wild rushing and jumping), while the control mice is unaffected (score 0).

Movie S2. Overview of interneuron migration in the cerebral cortex of control mice. Time lapse imaging of Dlx5/6-positive interneuron migration in control mice at E13.5. The movie length is 8 h and image interval is 2 min.

Movie S3. Tracking of interneuron migration in the cerebral cortex of control mice (related to Figure 3). Tracking of migrating cortical interneuron in control brain at the mi-cortical position.

Movie S4. Tracking of interneuron migration in the cerebral cortex of *Dlx5/6-Kif2a cKO* mice (related to Figure 3). Tracking of migrating cortical interneuron in mutant brain at mid-cortical position. Note that the number of stationary interneurons is higher than in control mice (Movie S3).
